# Optimizing Internet of Things Fog Computing: Through Lyapunov-Based Long Short-Term Memory Particle Swarm Optimization Algorithm for Energy Consumption Optimization †

**DOI:** 10.3390/s24041165

**Published:** 2024-02-10

**Authors:** Sheng Pan, Chenbin Huang, Jiajia Fan, Zheyan Shi, Junjie Tong, Hui Wang

**Affiliations:** School of Computer Science and Technology, Zhejiang Normal University, Jinhua 321004, China; person_er@zjnu.edu.cn (S.P.); ryenchen1@163.com (C.H.); jiajiaf@zjnu.edu.cn (J.F.); 1224412077@zjnu.edu.cn (Z.S.); 1375388402@zjnu.edu.cn (J.T.)

**Keywords:** predictive allocation, fog computing, internet of things (IoT), system stability, Lyapunov, LSTM, PSO

## Abstract

In the era of continuous development in Internet of Things (IoT) technology, smart services are penetrating various facets of societal life, leading to a growing demand for interconnected devices. Many contemporary devices are no longer mere data producers but also consumers of data. As a result, massive amounts of data are transmitted to the cloud, but the latency generated in edge-to-cloud communication is unacceptable for many tasks. In response to this, this paper introduces a novel contribution—a layered computing network built on the principles of fog computing, accompanied by a newly devised algorithm designed to optimize user tasks and allocate computing resources within rechargeable networks. The proposed algorithm, a synergy of Lyapunov-based, dynamic Long Short-Term Memory (LSTM) networks, and Particle Swarm Optimization (PSO), allows for predictive task allocation. The fog servers dynamically train LSTM networks to effectively forecast the data features of user tasks, facilitating proper unload decisions based on task priorities. In response to the challenge of slower hardware upgrades in edge devices compared to user demands, the algorithm optimizes the utilization of low-power devices and addresses performance limitations. Additionally, this paper considers the unique characteristics of rechargeable networks, where computing nodes acquire energy through charging. Utilizing Lyapunov functions for dynamic resource control enables nodes with abundant resources to maximize their potential, significantly reducing energy consumption and enhancing overall performance. The simulation results demonstrate that our algorithm surpasses traditional methods in terms of energy efficiency and resource allocation optimization. Despite the limitations of prediction accuracy in Fog Servers (FS), the proposed results significantly promote overall performance. The proposed approach improves the efficiency and the user experience of Internet of Things systems in terms of latency and energy consumption.

## 1. Introduction

In recent years, the proliferation of the Internet of Things (IoT) has led to a surge in smart mobile devices [1]. The advent of IPv6 technology has made everything being network-accessible a reality, and the commercialization of 5G technology further accelerated the increase in edge devices. With every grain of sand potentially having its own IP address, the deployment of numerous sensors to perceive the environment has become feasible. IoT analytics data show a growth from 6.1 billion global IoT device connections in 2017 to 14.4 billion in 2022, with an 18% increase in 2022 alone. Shockingly, by 2027, IoT connections might exceed 29 billion. However, this rapid expansion puts immense pressure on IoT infrastructure. The massive amount of data generated by numerous terminal sensing devices surpasses the processing capabilities of end-user devices. While cloud computing significantly eases this situation, its latency still fails to meet current user demands. Compared to the growth rate of terminal devices, the backbone network’s transmission speed has grown less than 10% annually [2]. Hence, public cloud server latency often exceeds 100 milliseconds [3], which is disadvantageous for delay-sensitive applications.

Recent advancements in fog computing underscore its revolutionary impact on traditional cloud computing. By redistributing computing, storage, and network resources to the edge and vicinity of IoT devices, fog computing effectively addresses the evolving demands of various applications. This paradigm shift is feasible because not all tasks necessitate the potent computing capabilities offered by cloud computing. Edge-based data processing and analysis have emerged as critical components of fog computing, furnishing real-time decision support and intelligence for IoT applications while optimizing latency and bandwidth.Simultaneously, much of the current research in fog computing revolves around real-time and near-real-time requirements [4]. The widespread adoption of this environment underscores the urgency for immediate data processing, emphasizing more than just response time. In autonomous driving systems, fog computing substantially reduces the transmission time between vehicles and remote cloud servers, achieving an ultra-low latency of less than 1 millisecond, as opposed to the 150 milliseconds latency observed in cloud computing [5,6]. This highlights the clear advantage of fog computing in enhancing the efficiency and responsiveness of real-time applications. Furthermore, fog computing, by elevating the Age of Information (AoI), significantly enhances the user experience in areas such as online gaming and Virtual Reality (VR). These applications fully exploit ultra-low latency, ensuring a seamless and immersive experience [7,8]. Consequently, future endeavors will concentrate on designing and modeling fog computing environments to achieve near-real-time response times, further aligning with the requirements of time-sensitive applications.

Task offloading involves transferring computational tasks from terminal devices or edge nodes to more powerful edge or cloud servers. This occurs when terminal devices or edge nodes lack sufficient computational resources or cannot complete specific tasks on time. The system must decide whether to transfer these tasks and related data to other devices or nodes for processing. While task offloading is a decision for the producer or the subsequent handler, resource allocation is a core concern for upper-level managers in distributed systems. They must assess the demands of various tasks and resources, considering the current state of available resources. However, while fog computing enhances task processing capabilities, it also faces potential latency increases due to the added burden from connecting more devices. In the pursuit of performance and speed, this can lead to device idleness and resource wastage.

In general models, total energy consumption for data processing includes both data transmission and processing energy. Data transmission energy depends on the channel status, which is influenced by the transmission time, power, and available bandwidth [9]. Higher transmission rates can be achieved by increasing power and reducing time but this increases the energy consumption. Balancing transmission power and time is key for minimal energy consumption, adjusting rates based on task latency requirements [10]. Computing energy is influenced by the computational capacity: increased resource input leads to exponential energy growth. Thus, allocating tasks to appropriate nodes, ensuring minimal resource input per unit while meeting latency requirements, is vital for energy efficiency [11].

Fog computing systems enhance decision-making and services by collecting and analyzing data from various devices, integrating contextual information. Even if some nodes or links fail, other nodes can continue operating. The focus in fog systems is on optimizing task processing through fog offloading and fog node resource allocation. This approach aims to improve overall resource utilization, thereby enhancing system performance and end-user experience. The three key considerations distilled from this are as follows:

(1) **Effective Resource Management in Fog Nodes or Cloud Servers:** Proper resource scheduling is crucial due to the inevitable presence of idle or underutilized nodes. Effective management can significantly amplify the impact of fog nodes.
(2) **Task Time Sensitivity and Priority:** Determining task priority is vital in current applications. Reasonable decision-making is necessary to prevent task stacking and enhance processing efficiency.
(3) **Dynamic Resource Demand in Fog Environments:** Fog systems often face dynamic changes in resource demand, which can cause instability. Appropriate systemic interventions help reduce overall energy consumption and ensure long-term stability.

**Figure 1:**
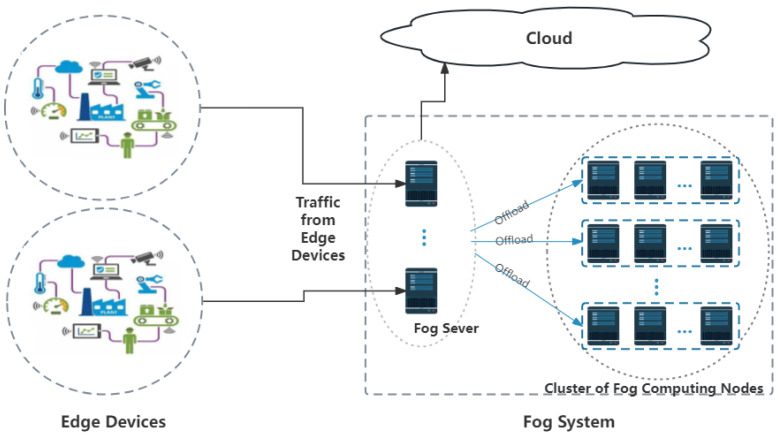
System architecture diagram.

This paper presents our perspectives on several key factors in fog computing, with the main contributions summarized as follows:

- **Dynamic Fog Federation Formation:** Considering the heterogeneity of fog device resources and the slower hardware update cycles compared to software, we propose a novel approach to establish dynamic fog federations. Utilizing heuristic algorithms, specifically Particle Swarm Optimization (PSO), we rapidly sense fog processor resources to form federations dynamically. These federations are tailored to each round of tasks, focusing on devices with high data correlation and low data heterogeneity.
- **Predictive Resource Allocation:** We introduce the concept of predictive resource allocation, driven by the unique data characteristics of end users. This approach dynamically adjusts model training based on outcomes to optimize resource allocation for task demands. It not only fulfills the need for tailored offloading solutions for different user profiles but also enhances overall performance through optimized prediction effectiveness.
- **System Stability and User Experience:** In consideration of long-term system stability, we analyzed the overall system’s stable conditions to enhance the user experience and quality on edge devices, thereby optimizing total energy consumption.
- **Model Implementation and Task Prioritization:** The model, depicted in Figure 1, illustrates how geographically proximate fog nodes form clusters. These clusters, possessing varied resources, are unified through virtualization technology and managed by a central, high-performance fog server. The central server orchestrates the workload distribution between fog servers and nodes, prioritizing tasks based on latency constraints. High-priority tasks are allocated to more capable fog servers, while medium-priority tasks are assigned later. To this end, we propose the Lyapunov-based Dynamic Long Short-Term Memory Predictive PSO Algorithm (LDLPSO) for the efficient management and prediction of task and resource allocation.

The structure of the paper is organized as follows. Section 2 introduces related work and research motivations. Section 3 describes the system model, including the transmission model, prediction model, priority assessment, energy consumption model, and algorithm design. Section 4 discusses the reasoning and feasibility of system energy consumption optimization. Section 5 presents the simulation results, analyzing the performance of the proposed allocation protocol compared to other allocation protocols. Section 6 outlines the conclusions of this paper.

## 2. Related Works

Since its inception in 2012 and formal definition in 2018 [12], fog computing has been the subject of extensive research efforts focused on enhancing system performance. The core of these efforts, as indicated in references [13,14,15,16], centers on optimizing task offloading and resource scheduling within fog computing systems. As demonstrated by references [10,17,18,19,20,21,22,23,24,25], these studies revolve around the stability of systems with limited computational resources and energy. Subsequently, within [1,2], there is a shift from passive to proactive, attempting to anticipate task request volumes and preparing for them in advance based on responses. Although these studies were published at different times, the direction of fog computing research is becoming more refined.

### 2.1. Works Focused on Optimizing Task Offloading

J. Flinn proposed that task offloading decisions should hinge on the resources accessible on mobile devices and the resources required for task execution [13]. Mainak Adhikari and his team’s research in fog computing, focusing on efficient IoT task scheduling and processing, inspired our work. They prioritized tasks based on deadlines and utilized multi-level feedback queues for appropriate device allocation [14]. Shichao Guan and colleagues developed an active hybrid offloading model for sustainable and heterogeneous offloading management in active cloudlets. This model, aimed at energy and QoS-aware heterogeneous offloading and resource allocation, adapts to various task and resource types [15]. It collaborates with cloud resources for partition and migration-based offloading, guided by task load and QoS demands. Xu Chen et al. investigated decentralized computing offloading games in mobile cloud computing environments, aiming for effective dispersed offloading via game-theoretic methods. Their approach focuses on the coordination of task offloading among mobile cloud users to enhance system performance and efficiency, reducing centralized management complexity and enabling self-organized decision-making among users [16]. The methods mentioned above revolve around task offloading and resource allocation. Multi-level feedback queues, lateral migration offloading, and decentralized management all provide references and inspiration for our subsequent work. However, their methods are limited by static resource availability, do not consider energy consumption, and are not suitable for delay-sensitive applications. On our study, we re-designed the way fog nodes provide computing resource services. The task-centric combination of fog computing allows for the dynamic adjustment of computing resources, considering low-power fog processors, thereby reducing the overall energy consumption.

### 2.2. Works Focused on Optimizing Energy Consumption

With the improvement in fog computing system performance, the issue of energy constraints on fog devices cannot be overlooked. Guowei Zhang and colleagues proposed an energy-minimizing task offloading algorithm centered on fairness. This approach focuses on three main aspects: the energy consumption of task offloading, the historical average energy consumption of fog nodes, and node priority [17]. They aim for fairness and minimized energy consumption in fog computing IoT environments by optimizing target fog nodes, transmission power, and sub-task sizes. Jianbo Du and colleagues developed the CORA algorithm, addressing computation offloading and resource allocation in hybrid fog–cloud computing systems. This algorithm aims to minimize the maximum weighted delay and energy consumption cost among user devices, ensuring user fairness and tolerable delay [18]. CORA coordinates offloading decisions and resource allocation, like computational resources, transmission power, and wireless bandwidth. Yu Qiu and colleagues focused on cost-effective optimization in FogC-IoMT (Fog Computing-based Internet of Medical Things) for healthcare monitoring [19]. They addressed challenges like time sensitivity, energy limitations, quality of service, and wireless constraints, breaking the problem into three sub-problems: medical task offloading, sub-channel allocation, and power distribution. They developed a sub-optimal, low-complexity algorithm to reduce energy consumption and transmission delay. Arash Bozorgchenani et al. proposed a method for multi-objective computation sharing in an energy- and delay-constrained mobile edge computing environment. By designing an evolutionary algorithm (NSGA2), they efficiently found the optimal balance between energy consumption and task processing delay, achieving minimal energy consumption and minimal processing delay for tasks [20]. The inspiration from the above works was to decompose complex problems into smaller ones, considering the joint optimization of energy consumption and task processing delay. However, their research did not take into account the performance differences between fog devices and overlooked the dynamic nature of fog computing systems.

Of course, to design a more robust system, it is necessary to consider the queue stability of joint optimization. Therefore, in our subsequent related work, we studied Lyapunov system control theory. Maganti Venkatesh et al. proposed an innovative deep learning mechanism, focusing on addressing workload balancing issues in fog computing [21]. By appropriately allocating workloads, they aimed to meet the QoS requirements of delay-sensitive IoT applications as much as possible. They considered workload distribution issues between fog nodes and the cloud, analyzing the stability of the IoT–fog–cloud queue scheme using Lyapunov drift and penalty theory. Karimiafshar et al. proposed the use of Lyapunov optimization techniques to ensure system stability while minimizing energy consumption and deadline misses in the deployment of fog computing resources in industrial IoT networks [10]. Yang Cai and Llorca, among others, made significant contributions to the efficient delivery of emerging distributed cloud architectures (such as fog and mobile edge computing) in real-time stream processing applications [22]. We also previously conducted a study on related topics. Huang et al. proposed a heuristic particle swarm optimization algorithm based on the Lyapunov optimization framework for resource scheduling and energy consumption optimization in fog computing [23]. They designed a novel queuing system that allows scheduling packets based on the current destination set, and developed the first fully decentralized, throughput, and cost-optimal multi-cast flow control algorithm using Lyapunov drift and penalty control theory. Their methods reflect considering the fog computing system from the perspective of system dynamics, which is achieved by controlling the stability of each optimization target queue. However, their research still deals with passive systems and can only be considered quasi real-time.

Subsequently, in order to improve the accuracy and scientific validity of our experiments, we scrutinized some experimental models, power consumption calculations, and fog server mode properties. Minghong Lin’s team addressed energy cost issues in data centers, proposing a model for dynamically adjusting the data center size to save costs. This model, based on an online algorithm for dynamic right-sizing, suggests shutting down servers during low-load periods to achieve significant cost savings, assuming perfect future workload prediction, which is a challenge in practical applications [24]. Y. Kim’s team used Monsoon power monitors to measure the power consumption of Galaxy Note smartphones with LTE chipsets across various mobile scenarios and Korean network operators. They fitted parameters to a typical CPU energy model, providing a basis for computational energy consumption and simulation in fog federations, highlighting the complexity and sensitivity to dynamic network states in their proposed strategy for balancing cost and latency in mobile edge computing environments [25].

### 2.3. Works Focused on Prediction

In order to continue to improve the speed in processing latency, people find another way toward predictive algorithm research, trying to analyze historical data to predict the amount of task data, and prepare for resource allocation in advance. Zhibo Li and colleagues developed a load prediction method for hybrid Mobile Edge Computing (MEC) servers using Bidirectional Long Short-Term Memory Networks (BILSTM). Known for its high predictive accuracy and minimal parameter configuration, this method is employed to optimize computing task offloading decisions, aiming to reduce task response times [2]. Despite enhanced predictive performance, their approach does not fully consider the time dependency of MEC server performance indicators and load levels, nor the resource energy consumption in practical applications. This suggests that, while improving prediction accuracy is crucial, balancing performance and resource efficiency in real-world deployment is also essential. Xin Gao and colleagues focused on dynamic offloading and resource allocation in multi-tier fog computing systems, based on traffic prediction. They introduced a stochastic network optimization problem aimed at minimizing the time-average power consumption while maintaining system stability [1]. Although their design reduced the average delay via a dual-layer fog structure, it did not fully address the general settings, task priorities, or resource availability, nor did it elaborate on how the prediction mechanism enhances system performance. The high dependency on prediction outcomes suggests a potential direction for future research.

Addressing the limitations identified in previous studies, this paper introduces the LDLPSO algorithm, designed to maximize the benefits of low-performance devices in a layered fog structure. It dynamically learns from historical data and assesses current states to predict data flows and prepare responses in advance, ensuring real-time task processing even under resource constraints. The optimized heuristic algorithm enables rapid real-time resource sensing and efficient resource allocation. It also reduces the overall energy consumption by integrating and utilizing resources effectively. Overall, there is still relatively little research on predictive offloading in fog computing systems. We compare this paper with the works in Table 1.

## 3. System Model

In this chapter, we will introduce the system’s topology, environment, and the technologies implemented to meet the required specifications. In Section 3.1, we discuss the system’s physical model and structure. In Section 3.2, we describe in detail the transmission model and the overall queueing model for tasks during transmission, including the evolution of predicted and actual queues. In addition, details related to the uplink delay are shown. Section 3.3 explains the implementation principles of the prediction model and its evolution in the system. Finally, in Section 3.4, we provide a detailed description of the energy consumption calculation details for the entire system. Of course, some related formula symbols can be analyzed in detail in Table 2.

Among these, the FS (Fog Server) is a core component of this system, and our algorithm will be deployed at this structural level. The FS is not only responsible for predicting the demand for computing resources but also for transmitting received data to the corresponding Fog Processor Nodes (CFCN) cluster. The following are the three tasks that the FS needs to perform:

(1) FS records this data as both the training and testing datasets for predictive functions.The dataset continuously updates with the ongoing reception by the FS. Therefore, during the dynamic process of predicting results, the model iterates gradually;
(2) Based on predicted values, we use the Particle Swarm Optimization algorithm (PSO) to rapidly search for suitable nodes or clusters of nodes and formulate a solution in advance;
(3) Finally, by comparing predicted results with actual results, adjustments to the allocation plan continue for those not in line with real values, while data that are in line are directly distributed to the corresponding Fog Processor Nodes (CFCN) cluster according to the plan.

In conclusion, the FS plays a crucial role in the model, dynamically predicting upcoming task loads in real time and allocating idle resources reasonably to cope with these tasks.

### 3.1. System Basic Elements

In the proposed system architecture, the fog computing layer adopts a two-layer structure to meet the data processing needs from *K* edge devices (represented as $I_{1},I_{2},\ldots ,I_{k}$). Edge devices generate data and transmit them to the fog layer wirelessly. The results are then subsequently obtained from it.

(1) Fog Layer 1: Comprising Fog Servers (FS), it is responsible for coordinating task assignment and scheduling. FS manages the status of each Fog Computing Node (FCN) in the next layer, recording the power consumption and computing capacity. When data from IoT devices reach this layer, the FS generates a cluster of Fog Processor Nodes (CFCN) specific to the tasks and allocates data to them for processing. The FS dynamically predicts the task load in real time and designs the allocated resource quantity;
(2) Fog Layer 2: Comprising N different FCNs, each represented as $F_{n}$ with n ranging from 1 to N. These nodes, arranged according to the fog processor $F_{s}$, dynamically combine into a cluster of Fog Processor Nodes (CFCN) to ensure a low power consumption and high-quality task completion. It is crucial to emphasize that the Fog Server (FS) serves as a critical link between edge devices and fog processors, which are typically connected through wired connections.

In summary, the Fog Server (FS) serves as a crucial link between edge devices and fog processors, which are typically connected through wired connections. The FS comprehensively understands the state of the fog layer, determining the optimal allocation of tasks among CFCNs to ensure the efficient utilization of computing resources and energy. Although we considered the cloud, this design did not allow us to study much about the offloading strategy between cloud and fog systems. The specific system model is illustrated in Figure 1.

### 3.2. Transmission Model and Task Queue

To enhance the realism of the transmission channel emulation, we incorporated a channel capacity calculation based on the Shannon capacity formula. This calculation quantifies the channel capacity between IoT devices and servers in Fog Layer 1. The length of each time slot for the wireless channel is denoted as τ, which remains constant within time slot *t* and may vary across different time slots. The wireless channel’s gain experiences decay over time, with decay power represented by $S_{k}\left(t\right)$. The transmission delay $C_{k,s}\left(t\right)$ is determined by the following formula [23]:

(1) $$C_{k,s}\left(t\right)=W\tau \log_{2}\left(1+\frac{P_{k,s(t)}S_{k,s(t)}}{W\sigma }\right)$$

where *W* signifies the wireless bandwidth; τ denotes the time slot length; $P_{k,s}\left(t\right)$ represents the transmitted power from IoT device *k* to $FS_{s}$ at time slot *t*; $S_{k,s}\left(t\right)$ indicates the fading gain of the wireless channel used by IoT device *k* to $FS_{s}$; and σ signifies the noise power.

In our system, a many-to-one relationship exists between user devices and fog servers. Task request intervals from user devices follow a random exponential distribution, consequently also making the service time provided by fog servers to user devices random and follow an exponential distribution. For example, when an IoT device sends a data packet to a fog server with no ongoing tasks, the server immediately handles the task. Fog server nodes primarily handle allocation and management, where task arrivals and processing are independent, adhering to the queuing theory M/M/1 model [26]. The queuing delay $W_{q}$ can be calculated using the following formula:

(2) $$W_{q}=\frac{\lambda }{\mu (\mu -\lambda )}$$

where λ represents the task arrival rate and μ represents the service rate.

Considering transmission delay further, we propose the transmission delay formula $\tau_{c}$ [23]:

(3) $$\tau_{c}=\frac{1}{\frac{C_{k,s}\left(t\right)}{\alpha_{k}\left(t\right)D_{k}\left(t\right)}-\alpha_{k}\left(t\right)\lambda_{k}\left(t\right)}$$

where $\tau_{c}$ represents transmission delay; $\alpha_{k}\left(t\right)$ represents the offloading ratio; $D_{k}\left(t\right)$ represents the average data size of device *k* tasks; and $C_{k,s}\left(t\right)$ represents transmission capacity.

This model comprehensively considers the impact of the offloading ratio and task data size on the transmission delay, providing crucial insights for task scheduling and transmission decisions in the system.

Between time slots *t*, we assume $I_{i}\left(t\right)$ ($T_{i}\left(t\right)\leq$Tmax for some constant Tmax) for the IoT device $I_{k}$ arriving at $F_{s}$. Tasks usually arrive at different time slots with varying processing sizes. Based on this, $F_{s}$ records the information sent by the task and employs an LSTM model algorithm to predict the future workload within a prediction window. $F_{s}$ deploys assigned tasks in advance based on the prediction, resulting in two types of queues on $F_{s}$:

(1) Prediction data: $P_{i,W_{k}}\left(t\right)$;
(2) Arrival data: $\lambda_{r}^{s}\left(t\right)$;
(3) Offload data: $\mu_{r}^{s}\left(t\right)$.

Actual tasks arriving at $F_{s}$ are arranged in the arrival queue and forwarded to the fog processor for task processing. Local processing resources are prioritized for the management of CFCNs.

Prediction Queue:

(4) $$A_{k,w}\left(t+1\right)=A_{k,w}\left(t\right)+\lambda_{r}^{s}\left(t\right)$$

Actual Queue:

(5) $$Q^{\left(R\right)}\left(t+1\right)=\max\left\{Q^{\left(R\right)}\left(t\right)+\lambda_{r}^{s}\left(t\right)-\mu_{r}\left(t\right)-U_{r}\left(t\right),0\right\}$$

### 3.3. Prediction Model

**Figure 2:**
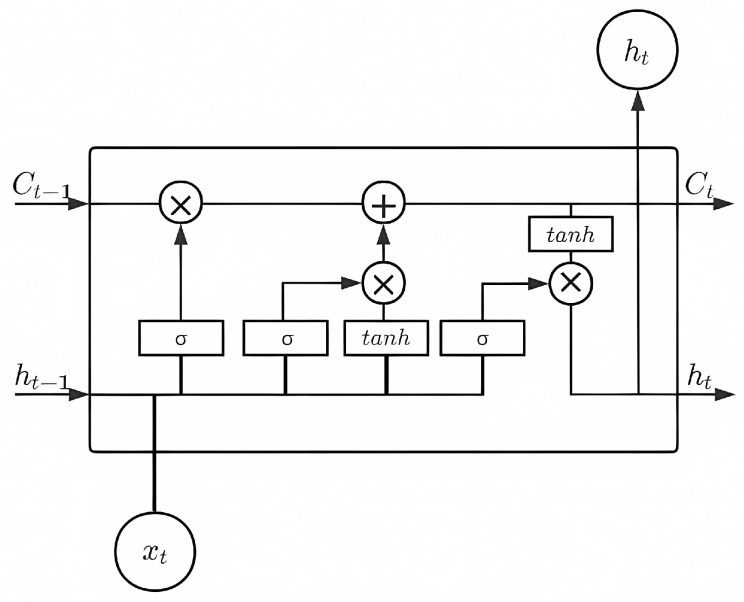
The structure of LSTM.

Based on the characteristics of user devices in heterogeneous networks and data analysis, it appears that a single device will periodically deliver packets, while delay-insensitive tasks do not require a high quality of service and only need to be completed within a given time frame. In this paper, we enhance LSTM by using sliding event windows for predicting and estimating resource requests of IoT devices to reduce energy loss while ensuring task completion. Long Short-Term Memory (LSTM) networks are a variant of Recurrent Neural Networks (RNNs) for processing sequential data, designed to address the long-term dependency problem prevalent in RNNs, all of which have a recurrent neural network module in the form of chains. In standard RNNs, this recurrent structural module only has a fairly common structure, such as a tanh layer. As the data are transformed while traversing the RNN, some information will be discarded at each time slot. After a period of time, the state of the RNN is almost devoid of any trace of the initial input. Therefore, when the conventional neurons are replaced by memory units, the LSTM will retain long-term information to some extent and solve the gradient explosion problem. The memory unit consists of three main controllers: the forgetting gate, input gate, and output gate. The model of LSTM is shown in Figure 2.

Data predictability typically requires a discernible pattern or trend, enabling statistical analysis and forecasting. Historical data are crucial for understanding system behavior, with data quality determining predictive model accuracy. Adequate computational resources are necessary for processing and analyzing large datasets. Stable systems or processes, with consistent behavior patterns, are more predictable. However, such stability is not always present in IoT edge computing. Certain sensors may operate stably over time, like those in environmental monitoring or surveillance. Encapsulating data formats and using dynamic LSTM models can stabilize predictions, optimizing performance and resource consumption. For constantly changing scenarios, a sliding window approach (initially set as Wk) reduces interference from large content variations. This dynamic training method sacrifices some training time for improved outcomes, applying predictions to resource pre-allocation when success rates reach a certain threshold.

### 3.4. Energy Model

The total power consumption P(t) of fog tiers in time slot t consists of the processing power consumption and wireless transmit power consumption, where the processing work number, in turn, contains the distribution power consumption of the fog server and the computational power consumption of the fog processor, given a local CPU with frequency f.

In the previous section, the task queue in the energy consumption model for task processing follows the M/M/1 pattern. The local computational delay $\tau_{i}$ can be expressed using the following formula:

(6) $$\tau_{i}=\frac{\lambda_{i}\left(t\right)\left({\sigma_{i}^{F}}^{2}+D_{i}^{2}\right)}{2(F_{i}^{2}-\lambda_{i}\left(t\right)F_{i}D_{i})}+\frac{D_{i}}{F_{i}}$$

where λ denotes the probability of task arrival; σ represents the standard deviation of the task size; and $F_{i}$ denotes the computational resources of i. The total energy consumed in performing a task is, therefore, represented as $P_{i}^{l}$:

(7) $$P_{i}^{l}=\sum_{i=1}^{N}p_{i}\left(t\right)$$

Therefore, in our system, delay-sensitive tasks are allocated processing based on computational requirements: a perception of the current local resources is made, and if there is the capacity to handle them, computational offloading is not considered. Otherwise, the task data that need to be processed are allocated according to the current resource status of the fog node by the control node, and offloaded to the fog processor layer for processing, as shown in Figure 1.

The total energy consumption of delay tasks can be expressed as:

(8) $$P\left(t\right)=\sum_{s=1}^{S}p_{s}\left(t\right)+\sum_{i=1}^{N}p_{i,j}^{c}\left(t\right)+\sum_{i=1}^{N}\left(1-\alpha \right)p_{i}\left(t\right)$$

where $p_{i}\left(t\right)$ represents the energy consumed by fog node s. Y. Kim et al. measured the power consumption of a Galaxy Note smartphone equipped with an LTE chipset using a Monsoon power monitor for three network operators and various mobile scenarios in South Korea by fitting the parameters (κ, ϕ, ρ) to a typical CPU energy model in [16], where δt denotes the duration of one time slot in the 15th second, as shown below [25].

(9) $$E_{s}\left(s^{i}\left(t\right)\right)=\left(\kappa {\left(s^{i}\left(t\right)\right)}^{\varphi }+\rho \right)\Delta t.$$

The measured energy consumption of the LTE and Wi-Fi networks was 2605 mJ/s and 1225 mJ/s, respectively, and the CPU energy parameter was (κ, ϕ, ρ) = (0.33, 3.00, 0.10) [24]. So, as described in [27], $p_{i}\left(t\right)$ can be expressed as

(10) $$p_{s}\left(t\right)=\tau_{s}\zeta {\left(F_{s}\left(t\right)\right)}^{3}$$

where ζ is a parameter depending on the deployed hardware and is measurable in practice.

In this paper, $P_{i,j}^{c}\left(t\right)=\tau_{c}p_{i}^{c}\left(t\right)$ is defined as the energy consumption of the device *i* transmitting tasks to *j*. *M* represents the set of IoT nodes, and N represents the set of fog nodes.

## 4. Problem Formulation

The optimization of total energy consumption is a key concern in this study. We adopt the Lyapunov approach to optimize total energy consumption. Ensuring the condition that the queue is stable, the entire optimization problem can be transformed into a Lyapunov optimization problem. That is, to guarantee the minimum average energy consumption per time slot, it can be represented as:

(11) $$p1:\&\lim_{T\to \infty }\frac{1}{T}\sum_{t=0}^{T-1}P_{a}\left(t\right)$$

(12) $$s.t.\;\lim_{T\to \infty }\frac{1}{T}\sum_{t=0}^{T-1}\sum_{i=1}^{N}F_{i,s}\left(t\right)<F_{s}$$

(13) $$\lim_{T\to \infty }\frac{1}{T}\sum_{t=0}^{T-1}\sum_{i=1}^{N}C_{i,j}\left(t\right)<C_{j}$$

(14) $$0\leq p_{i}\left(t\right)\leq P_{i}^{b}$$

(15) $$0\leq F_{ij}\left(t\right)\leq F_{s}$$

(16) $$0\leq C_{ij}\left(t\right)\leq C_{j}$$

The objective of P1 is to reduce the long-term average energy consumption across all IoT devices [28], necessitating full task offloading to computing nodes in the fog network, as denoted by $F_{a}$. Decision variables include the partition of computing resources for offloading $\alpha_{i}\left(t\right),F_{i}\left(t\right)$, transmission power per time slot $P_{i}\left(t\right)$, and computational resources $F_{s}$ of the computing layer nodes, with i∈N. These variables are influenced by the task size, the required processing latency, and the objective function, under constraints of the channel bandwidth, computational resources (P1 and $F_{s}$), and IoT nodes’ transmission power. It is assumed that all time slots are equal in length.

P1 also considers limitations on the computing resources allocated by device i (15) and device i’s transmission speed (16). Over time, the allocated resources must be less than the device’s capacity, which is constrained by $F_{s}$. Considering the device’s transmission capability, the bandwidth occupied by tasks should be less than the total channel bandwidth, ensuring energy usage remains below the battery’s total capacity. Local processing of tasks only accounts for energy consumption.

### 4.1. Setting Up Lyapunov Virtual Pairs of Columns

In Lyapunov optimization, the satisfaction of long-term average constraints is equated to the rate stability of the virtual queue. To be more precise, a virtual queue is introduced to replace the computation resource constraint at edge node (11), and G(t) represents the random process of the virtual queue length at time slot t. The channel constraint is denoted by C(t), and the explicit form of G(t) and C(t) can be represented as follows:

(17) $$G\left(t+1\right)-G\left(t\right)=\max\left(\sum_{i=1}^{N}F_{i,s}\left(t\right)-F_{s},-G\left(t\right)\right)$$

(18) $$H\left(t+1\right)=\max\left(H\left(t\right)+\sum_{i=1}^{N}C_{i,j}\left(t\right)-C_{j},0\right)$$

This study assumes a stable environment, with a consistent transmission power from the charging device. The estimated value of the charging amount $P_{i}^{opt}\left(t\right)$ can be expressed as:

(19) $$P_{i}^{opt}\left(t\right)=\frac{1-e^{-\psi (t)d}}{{\left(\psi (t)d\right)}^{2}}$$

where d denotes the distance between the sender and the receiver, and ψ denotes the decay factor, often determined by the signal frequency and medium characteristics. If the virtual queue is rate-stable, only $\lim_{T\to \infty }\frac{A(T)}{T}$ can meet constraint (11) according to the definition in [29]. Similarly, we can derive channel backlog virtual queue B, delay backlog virtual queue C, and edge node energy backlog virtual queue D. The proof is as follows:

First, we prove the stability of the virtual queue:

(20) $$G\left(t+1\right)-G\left(t\right)=\max\left(\sum_{i=1}^{N}F_{i,s}\left(t\right)-F_{s},-G\left(t\right)\right)$$

For t∈(0,1,2,…,T−1), there exists

(21) $$\lim_{T\to \infty }\frac{G(T)-G(0)}{T}\geq \lim_{T\to \infty }\frac{1}{T}\sum_{t=0}^{T-1}\sum_{i=1}^{N}F_{i,s}\left(t\right)-F_{s}$$

If A(0) = 0, then

(22) $$\lim_{T\to \infty }\frac{G(T)}{T}=0$$

Hence, there is:

(23) $$\lim_{T\to \infty }\frac{1}{T}\sum_{t=0}^{T-1}\sum_{i=1}^{N}F_{i,j}\left(t\right)\leq F_{j}$$

Therefore, queue A is stable, and similarly, virtual queue B(t) is stable, yielding:

(24) $$\lim_{T\to \infty }\frac{1}{T}\sum_{t=0}^{T-1}\sum_{i=1}^{N}C_{i,j}\left(t\right)\leq C_{j}$$

After proving the above, we can translate problem P1 into problem P2.

### 4.2. Constructing the Lyapunov Function

In this subsection, the specific Lyapunov derivation process and the translation of problem P1 into problem P2 will be shown, as represented by the following equations:

(25) P2:limT→∞1T∑t=0T−1Pa(t)s.t. G(t) is rate stables.t. H(t) is rate stables.t. (14)–(16)

Equations (14)–(16) set up the virtual queue vector, with the system state represented as θ(t):

(26) Θ(t)=[G(t),H(t)]

(27) $$\begin{array}{cc} L(\theta (t)) & \begin{array}{c} =\frac{1}{2}\sum_{i=1}Q_{i}{\left(t\right)}^{2} \end{array}\\ \; & =\frac{1}{2}\left(G{\left(t\right)}^{2}+H{\left(t\right)}^{2}\right) \end{array}$$

(28) Δθ(t)=E(L(θ(t+1))−L(θ(t))∣θ(t))

When the rate in each queue is stable, problem P2 can be transformed into problem P3, because the original problem with long-term average objectives and constraints can be approximately transformed into a problem with drift plus penalty. In this paper, it is assumed that:

(29) $$P\left(t\right)=\sum_{t=0}^{T-1}P_{a}\left(t\right)$$

Based on the Lyapunov drift plus penalty algorithm, it can be converted into problem P3 as shown below:

(30) $$\begin{array}{cc} P3:\; & \min\Delta \theta (t)+VE(P(t)|\theta (t))\\ s.t.\; & \left(12),(13\right) \end{array}$$

where V represents the weight of the objective function, and Δθ(t) represents the drift of the queue, i.e., the stability of the queue. The following shows the specific form of A to prepare for the upper bound of P4. First, we show $G{\left(t+1\right)}^{2}-G{\left(t\right)}^{2}$:

(31) $$\begin{array}{cc} \; & \begin{array}{c} G{\left(t+1\right)}^{2}-G{\left(t\right)}^{2} \end{array}\\ \; & =\max{\left(G\left(t\right)+\sum_{i=1}^{N}F_{i,s}\left(t\right)-F_{s},0\right)}^{2}-G{\left(t\right)}^{2}\\ \; & \leq 2G\left(t\right)\left(\sum_{i=1}^{N}F_{i,s}\left(t\right)-F_{s}\right)+{\left(\sum_{i=1}^{N}F_{i,s}\left(t\right)-F_{s}\right)}^{2}\\ \; & \leq 2G\left(t\right)\sum_{i=1}^{N}F_{i,s}\left(t\right)-2G\left(t\right)F_{s}+{\left(\sum_{i=1}^{N}F_{i,s}\left(t\right)\right)}^{2}\\ \; & -2F_{s}\sum_{i=1}^{N}F_{i,s}\left(t\right)+F_{s}^{2}\\ \; & \leq 2\left(G\left(t\right)-F_{s}\right)\sum_{i=1}^{N}F_{i,s}\left(t\right)+{\left(\sum_{i=1}^{N}F_{i,s}\left(t\right)\right)}^{2}+F_{s}^{2}\\ \; & \leq 2\left(G\left(t\right)-F_{s}\right){\sum_{i=1}^{N}F_{i,s}\left(t\right)+\left(N+1\right)F_{s}}^{2}\\ \; & =D_{1}+2\left(G\left(t\right)-F_{s}\right)\sum_{i=1}^{N}F_{i,s}\left(t\right) \end{array}$$

Here, the fixed value is represented by $D_{1}$:

(32) $$D_{1}=\left(N+1\right)F_{s}^{2}$$

Similarly, the virtual queue B under bandwidth constraints can be represented as:

(33) $$H{\left(t+1\right)}^{2}-H{\left(t\right)}^{2}=D_{2}+2\left(H\left(t\right)-C_{j}\right)\sum_{i=1}^{N}C_{i,j}\left(t\right)$$

where $D_{2}$ represents a fixed constant and is expressed by the following formula:

(34) $$D_{2}=\left(N+1\right)C_{j}^{2}$$

Problem P3 can be expanded to problem P4:

(35) $$\begin{array}{cc} P4:\; & \min\;VP\left(t\right)+\left(G\left(t\right)-F_{s}\right)\sum_{i=1}^{N}F_{i,s}\left(t\right)+\left(H\left(t\right)-C_{j}\right)\sum_{i=1}^{N}C_{i,j}\left(t\right)\\ \; & s.t.\;(14),(15),(16) \end{array}$$

To prove that virtual queues G and H are stable within the context of problem P4, where P4 has an upper bound constant C, the proof involves demonstrating that these queues do not grow indefinitely over time. This is typically achieved by showing that the long-term average inflow rate to each queue is less than or equal to its outflow rate. By establishing this condition, it can be inferred that the queues will remain bounded and thus stable, ensuring that the system operates within its defined constraints. The proof is as follows:

(36) $$\begin{array}{cc} L(\theta (t+1))-L(\theta (t))+VP(t)\leq C\\ \;L(\theta (t+1))-L(\theta (t)) & \leq C\\ L(\theta (T))-L(\theta (0)) & \leq TC \end{array}$$

This is obtained by making L(θ(0))=0:

(37) $$\begin{array}{cc} \frac{1}{2}\left(G{\left(t\right)}^{2}+H{\left(t\right)}^{2}\right) & \leq TC\\ G\;{\left(t\right)}^{2} & \leq 2TC\\ G\left(t\right) & \leq \sqrt{2TC} \end{array}$$

Thus, continuing the derivation yields:

(38) $$\lim_{T\to \infty }\frac{G(T)}{T}\leq \lim_{T\to \infty }\frac{\sqrt{2TC}}{T}=0$$

The following conclusions can then be drawn:

(39) $$\lim_{T\to \infty }\frac{G(T)}{T}=0$$

Thus, we can assume that the queue is stable, and we will use an approximate analysis to explore the upper bound of problem P4.

### 4.3. Upper Bound Analysis

The reasoning in the previous section proved that problem P4 to be solved has an upper bound, and we will then solve it for the exact value of this upper bound. First, it is necessary to prove the upper bound by substituting the computational drift into Equation (40):

(40) $$\lim_{T\to \infty }\frac{1}{T}\sum_{t=0}^{T-1}P^{\prime }\left(t\right)\leq P*+\zeta$$

where the specific deviation ζ is as follows:

(41) $$\zeta =\frac{\left(N^{2}+1\right)\left({F_{j}}^{2}+{C_{j}}^{2}\right)+2NG_{\max}F_{j}+2NH{\;}_{\max}C_{j}}{2V}$$

According to the process of solving queuing network problems using the Lyapunov optimization method, the queue length is usually regarded as a random process. Then, the Lyapunov function is constructed to estimate the expected growth rate of the queue length, and the queue length is controlled in a certain range. When the constructed function meets certain conditions, it can be proved by stability theory that the queue length can be controlled within a certain range, and the optimal solution can be obtained. Of course, these results are subject to deviation. For the problem of deviation range, a penalty term is introduced into the Lyapunov function to limit the fluctuation of the queue length to a certain range, so that the problem can obtain an optimal solution within a certain deviation range. This approach is called the Lyapunov drift-plus-penalty method [30]. The specific proof is as follows:

(42) $$\begin{array}{cc} \; & \Delta \Theta^{\prime }\left(t\right)+VE\left(P^{\prime }\left(t\right)|\Theta \left(t\right)\right)\\ \; & \leq \frac{D_{1}+D_{2}}{2}+VP^{\prime }\left(t\right)+\left(G\;\left(t\right)-F{\;}_{j}\right)\sum_{i=1}^{N}{F^{\prime }}_{i,s}\left(t\right)\\ \; & +\left(H\;\left(t\right)-C_{j}\right)\sum_{i=1}^{N}C_{i,j}^{\prime }\left(t\right)\\ \; & \leq \frac{D_{1}+D_{2}}{2}+VP*+N\left(G_{\max}F_{j}+H_{\max}C_{j}\right) \end{array}$$

Subtracting $\Delta \Theta^{\prime }\left(t\right)$ from both sides and dividing by V at the same time reduces the original equation to:

(43) $$\begin{array}{cc} \; & \lim_{T\to \infty }\frac{1}{T}\sum_{t=0}^{T-1}P^{\prime }\left(t\right)\\ \; & \leq \frac{D_{1}+D_{2}}{2V}+P*\\ \; & +\frac{N(G_{\max}F_{j}+H_{\max}C_{j})}{V}-\frac{\Delta \Theta^{\prime }\left(t\right)}{V} \end{array}$$

The next expansion of $\Delta \Theta^{\prime }\left(t\right)$ yields the upper bound ζ

(44) $$\begin{array}{cc} \; & \lim_{T\to \infty }\frac{1}{T}\sum_{t=0}^{T-1}P^{\prime }\left(t\right)\\ \; & \leq \frac{D_{1}+D_{2}}{2V}+P*\\ \; & +\frac{N(G_{\max}F_{j}+H_{\max}C_{j})}{V}-\frac{\Delta \Theta^{\prime }\left(t\right)}{V}\\ \; & =\frac{D_{1}+D_{2}}{2V}+P*+\frac{N(G_{\max}F_{j}+H_{\max}C_{j})}{V}\\ \; & -\lim_{T\to \infty }\frac{L\left(\theta^{\prime }\left(T-1\right)\right)-L\left(\theta^{\prime }\left(0\right)\right)}{VT}\\ \; & =P*+\frac{{\left(N+1\right)}^{2}{F_{j}}^{2}+{\left(N+1\right)}^{2}{C_{j}}^{2}}{2V}+\frac{NG_{\max}F_{j}}{V}\\ \; & =P*+\frac{\left(N^{2}+1\right){F_{j}}^{2}+{C_{j}}^{2})+2NG_{\max}F_{j}+2NH_{\max}C_{j}}{2V} \end{array}$$

Overall, we initially precisely defined the problem, aiming to optimize the total energy consumption of the Internet of Things (IoT) system using the Lyapunov method. Subsequently, we systematically established the Lyapunov function, transforming problem P1 into P3 and introducing a virtual queue that encompasses aspects such as computing resources, channels, delays, and energy. By penalizing the Lyapunov drift, we conducted an upper-bound analysis of problem P4, providing crucial insights for the theoretical analysis of problem solutions. This establishes a theoretical foundation for the algorithm design, the construction of the fitness function, and the long-term operation of the system in the subsequent fifth section.

## 5. Algorithm Design and Numerical Results

This section outlines and analyzes the algorithm. The Fog Servers (FS) pre-processed data from edge or IoT devices, integrating types, volumes, and pre-processing times. A predictive model was then built using Pytorch, trained on 90% of the data, with the remaining 10% being used for model validation. A key consideration was selecting data volumes, and setting Wk to 4–20% of the total time slot. The predictive window size was adjusted based on feedback from each prediction, optimizing the results without affecting the allocation. At Algorithm 1, The algorithm details the construction of a sliding prediction model, predicting offloading data, and adjusting the prediction accuracy by using some training costs as a trade-off. PSO-based resource allocation is described in the Algorithm 2, also addressing system stability requirements.
**Algorithm 1** Prediction and Allocation Algorithm using LSTM and PSO.**Require:** $W_{k},Q\left(t\right),\lambda_{r}^{s}\left(t\right),U_{r}\left(t\right),P_{c},P_{i},P_{s},T_{MAX}$**Ensure:** $0<t<T_{MAX}$1:Initialize LSTM model with parameters2:Initialize PSO algorithm for processor allocation3:**for** t=0 to $T_{MAX}$ **do**4:    Update $\left\{\lambda_{r}^{s}\left(t\right)\right\}$, $\left\{A_{w}\left(t\right)\right\}$5:    $Q\left(t\right)=Q\left(t-1\right)+\lambda_{r}^{s}\left(t\right)-\mu_{r}\left(t\right)-U_{r}\left(t\right)$           ▹ update task queue6:    **if** $t\geq W_{k}$ **then**7:        $A_{w}\left(t\right)=$ LSTM Prediction $\left(Q\left(t-W_{k}:t-1\right)\right)$      ▹ predict using LSTM8:    **end if**9:    $U_{r}{\left(t\right)}^{\prime }=$ PSO Allocation$\left(A_{w}\left(t\right)\right)$            ▹ PSO based allocation10:    **if** $A_{w}\left(t\right)\approx \lambda_{r}^{s}\left(t\right)$ **then**11:        $U_{r}\left(t\right)=U_{r}{\left(t\right)}^{\prime }$                  ▹ use predicted allocation12:    **else**13:        $U_{r}\left(t\right)=$ PSO Allocation$\left(\lambda_{r}^{s}\left(t\right)\right)$          ▹ reallocate based on actual data14:    **end if**15:    Execute allocation based on $U_{r}\left(t\right)$16:    Update prediction accuracy $E_{r}$17:    **if** $\left(E_{r}<60\%\right)$ and $\left(W_{k}<20\%\times T_{max}\right)$ **then**18:        $W_{k}+=5$              ▹ increase window size if accuracy low19:    **else if** $\left(E_{r}\geq 60\%\right)$ and $\left(W_{k}>5\%\times T_{max}\right)$ **then**20:        $W_{k}*=0.9$             ▹ decrease window size if accuracy high21:    **end if**22:**end for**

**Algorithm 2** Allocation Algorithm Based on Lya-PSO.
**Require:** 

$I_{k},A_{k}\left(t\right),P\left(t\right),F\left(k\right),F\left(j\right),F\left(n\right),P_{k},P_{c},P_{n},U_{r}\left(t\right)$

**Ensure:** 

$0<F\left(k\right)<F{\left(k\right)}_{MAX},0<F\left(n\right)<F{\left(n\right)}_{MAX},0<C^{k,n}<C_{MAX}^{k,n}$

1:// Initialize task allocation process2:**for** i=1 to *K* **do**3:    Initialize position $X_{k}$ and velocity $V_{k}$ for particle *k*4:    Define objective function $\min\;VP\left(t\right)+\left(G\;\left(t\right)-F_{s}\right)\sum_{k=1}^{N}F_{k,s}\left(t\right)+\left(H\;\left(t\right)-C_{j}\right)\sum_{k=1}^{N}C_{k,j}\left(t\right)$5:    Evaluate particle *k* and set pBest6:    Update parameters = $X_{k}$7:
**end for**
8:// Verification and Allocation9:**if** $X_{k}==\mathrm{null}$ and F(j)≠null **then**10:    **for** n=1 to *N* **do**11:        **if** $F_{n}$ is free **then**12:           Allocate an idle fog processor13:           Recursive: $A_{k}\left(t\right)+=\mathrm{Allocation}\;\mathrm{algorithm}\left(I_{k}\right)$ ▹ Find the best matching fog processor group14:        **end if**15:    **end for**16:
**end if**
17:**return** $P*\left(t\right),A_{k}^{*}\left(t\right)$                  ▹ Return the optimal allocation


In this section, the performance of the proposed LLPSO algorithm is evaluated through numerical simulations. The test was conducted on a Windows 10 system with a 2.3 GHz Intel Core i5-8300H processor and 24 GB 2667-MHz DDR4 memory. The simulation environment was built using Python 3.10. The section begins with the basic setup of the simulation environment and then presents results and analyses of the algorithm’s performance under different latency requirements for tasks.

### 5.1. Basic Settings

In the simulation, a fog computing system with 20 users, 50 computing nodes, and one control node was modeled. Each control node corresponded to computing nodes, all active with varying total resources. Each node had different available resources, and available memory and CPU for dynamically processing change when offloading tasks.

Twenty users were randomly simulated to send task requests over an hour. The bandwidth between users and fog control nodes was set at 10 Mb/s, with wired connections to fog computing nodes. Tasks were uploaded to edge devices, which decided on local processing or offloading. Users could also connect directly to fog servers. Users and edge devices, which were treated as users, had initial CPU capacities of 500 Mhz and 500 M of memory. Fog server nodes had higher bandwidths of 2 GHz and 4 G RAM. Processor nodes started with 300 Mhz CPUs and 300 M RAM. The communication interference and distances between users and computing nodes were constant, while noise between users and the cloud varied. Task sizes ranged from 1–10 M, and the algorithm’s inertia weight (w) was set at 0.6.

### 5.2. Comparative Analysis of Prediction Algorithms

Firstly, we present a partial data comparison graph between the optimized prediction algorithm and real data. Prediction, as a task, is inherently challenging, considering it deals with unknown events. The effectiveness of predictions becomes more evident when the data exhibit certain patterns that can be predicted. In this experiment, we initially conducted a comparison of predictions involving standalone LSTM, LSTM with a fixed sliding window mode, and LSTM with a dynamic sliding window.

**Figure 3:**
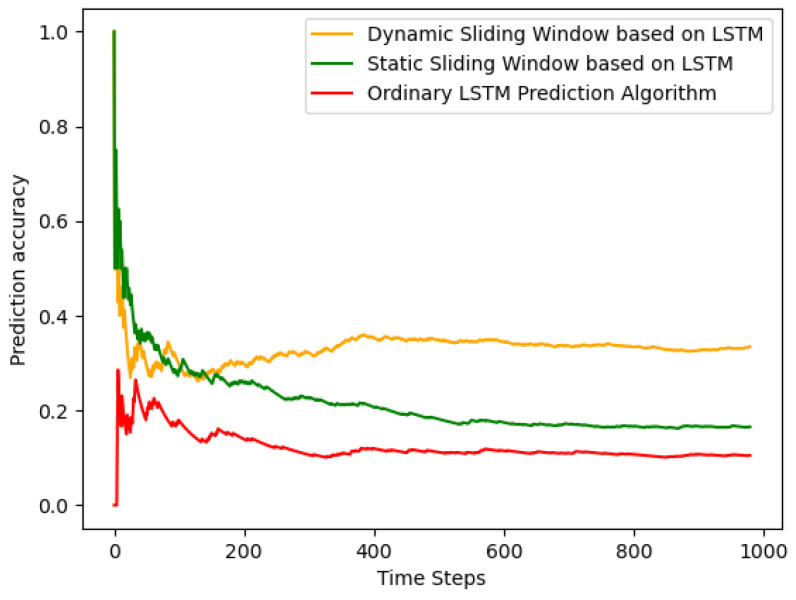
Accuracy trend at each time slot.

Firstly, in Figure 3, you can see a comparative graph of the prediction accuracy for each time slot among the three prediction methods. In general cases, the prediction started with failure, resulting in a starting point at 0 and 1. At this point, the simulation represented extreme conditions where user data were continuously changing, and this data variation was influenced by the bandwidth, transmission rate, and processing latency. According to the evaluation criteria in this paper, it was observed that the dynamic window, WK, which adjusted its accuracy gradually with model training, exhibited a relatively higher precision.

**Figure 4:**
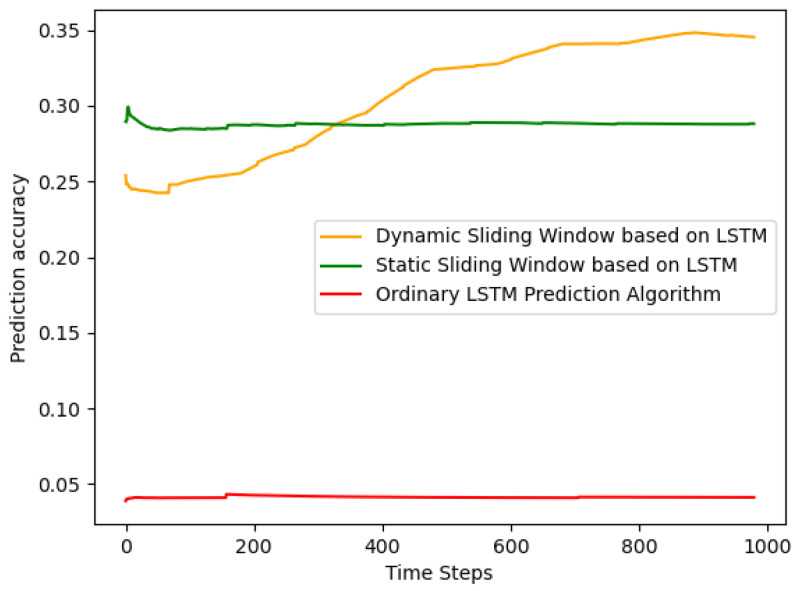
Training time under each time slot.

Figure 4 illustrates the training time comparison for the three algorithms in each time slot. Firstly, the general LSTM model could be used directly after training; thus, it did not consume a significant amount of training time. The training time for LSTM optimized under the dynamic sliding window was initially faster than that optimized using the static window training, but later it took longer. The comparison between dynamic and static arose because the algorithm adjusted during continuous operation, discovering that investing some training time appropriately could enhance accuracy. Secondly, the training time was determined by the channel rate as the lower limit and when to use these data as the upper limit.

In summary, in this design, the prediction method of LSTM optimized under the dynamic sliding window, compared to LSTM optimized under the static window, improved our accuracy by approximately 3.28 times and the precision by over 18.02%. Compared to the regular LSTM prediction method, our accuracy increased by 1.422 times, and the precision improved by over 7.72%.

### 5.3. Evaluation with Different Delay Requirement Task

We evaluated the performance of various algorithms for tasks with different latency requirements and also presented a comparison of delays after incorporating prediction algorithms. Additionally, we assessed the system performance, including the impact of node expansion on system performance, tasks with the same number of nodes but different arrival rates, and a comparison of error rates for tasks with different arrival rates.

**Figure 5:**
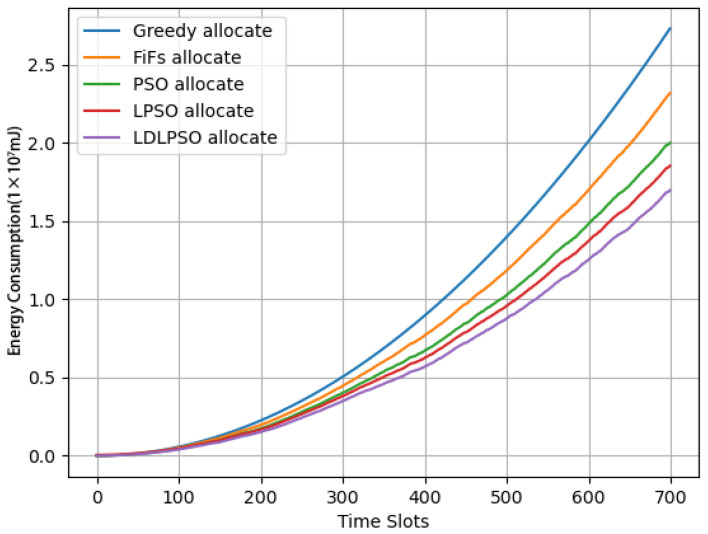
Total time consumption of various tasks.

**Figure 6:**
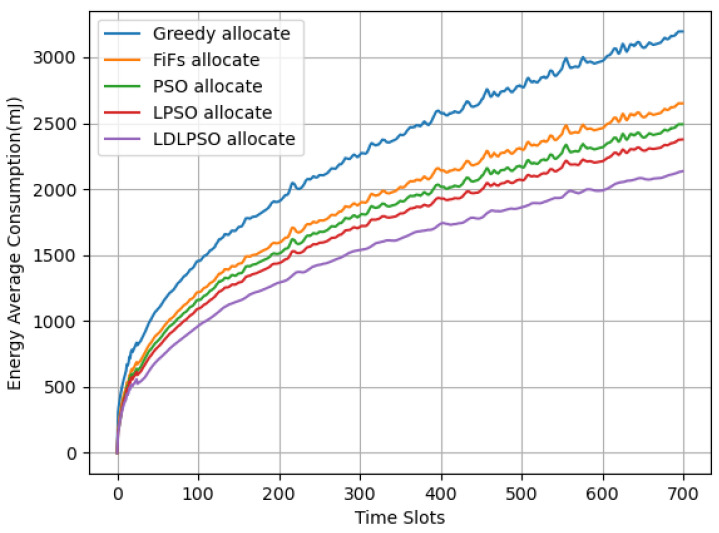
Average energy consumption graph of time slots.

In Figure 5, we can observe the total energy consumption variation for different algorithms in each time slot, and Figure 6 illustrates the average energy consumption variation in different time slots. Among them, the improved LDLPSO algorithm performed the best, followed by the LPSO algorithm, the original PSO algorithm, the First-Come-First-Served (FiFs) algorithm, and the Greedy algorithm. The reason for this phenomenon is that our proposed LDLPSO algorithm takes into account the stability of the entire system queue, making its line relatively clear with smaller fluctuations. Although the graph appears relatively stable, the actual numerical changes are significant. After comparison, it can be concluded that the LDLPSO algorithm saves an average of 9.44% in energy consumption compared to the LPSO algorithm and 32.73% compared to the most energy-consuming algorithm, i.e., the Greedy algorithm.

**Figure 7:**
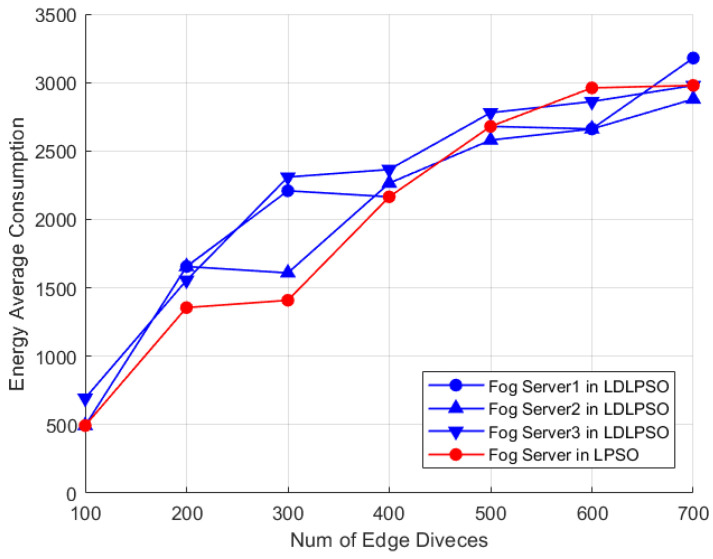
Performance of two algorithms: average energy consumption of FS with increasing number of nodes.

**Figure 8:**
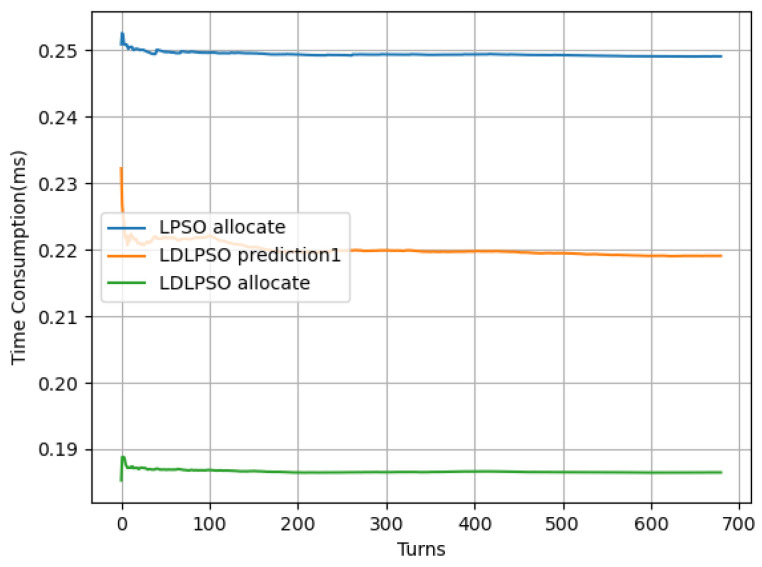
Performance of two algorithms: round averaging latency distribution.

In Figure 7 and Figure 8, we further compare the energy consumption and latency between the LDLPSO and LPSO algorithms for additional research. On one hand, although the Full System (FS) energy consumption increased, the overall average energy consumption did not, thanks to its dynamic combination fog processor scheme, which was designed for tasks. Simultaneously, due to the effect of pre-allocation, our allocation speed increased by 1.25 times compared to the original speed. On the other hand, with the increase in the number of nodes, the variation in our average energy consumption remained relatively stable. As for the other strategies, the First-Come-First-Served (FiFs) strategy led to queue congestion and increased task delays, requiring more computational and communication resources to meet the deadline and consequently increasing energy consumption. Finally, the Greedy algorithm consumed more energy in an attempt to complete all tasks as quickly as possible.

**Figure 9:**
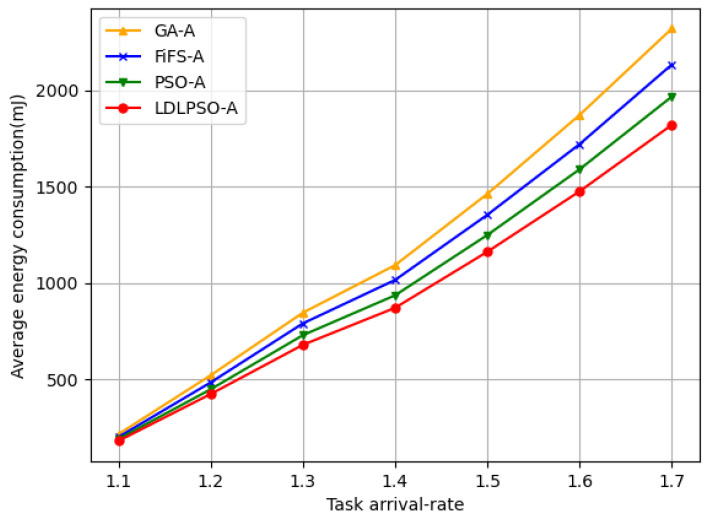
The energy consumption chart for different algorithms under different arrival rates.

**Figure 10:**
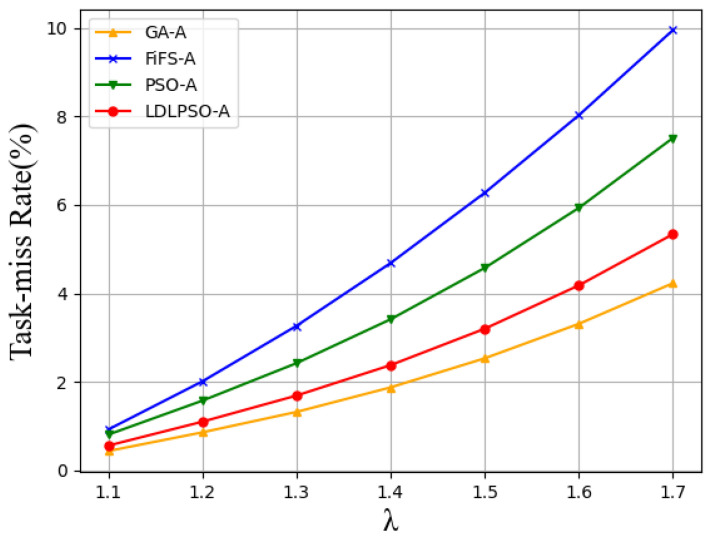
The task-miss rate for different algorithms under different arrival rates.

In Figure 9, we can observe that, even with the same arrival rate, our proposed LLPSO algorithm still performed the best. This is because the combination of Lyapunov-based system control and LSTM-based task prediction, while maintaining queue stability, significantly reduces the queue time and lowers system energy consumption. In contrast, the other three algorithms focus solely on resource allocation without dynamic optimization. Additionally, we compared the performance of different algorithms under different task arrival rates with the same number of user nodes, as shown in Figure 10. In these comparisons, the Greedy algorithm outperformed the LDLPSO algorithm. This is because the Greedy algorithm allocates a significant amount of resources and energy, thereby increasing the task completion rate. On the other hand, compared to LDLPSO, the performance of the other two algorithms in terms of task completion rate is relatively poor. LDLPSO can effectively utilize available resources, prioritize optimizing task allocation, and ensure tasks are completed on time. However, the other two algorithms lack flexibility in resource utilization and task allocation, thus they cannot fully leverage the available resources to meet task demands, resulting in a lower task completion rate.

In addition, we also compared the performance of different algorithms under different task arrival rates with the same number of user nodes. In these comparisons, the Greedy algorithm outperformed the LLPSO algorithm. This is because the Greedy algorithm invests significant resources and energy, resulting in a higher task completion rate. On the other hand, the other two algorithms performed relatively poorly in terms of the task completion rate compared to LDLPSO. LDLPSO utilizes the available resources efficiently and prioritizes optimal task allocation to ensure timely task completion. It allocates a substantial amount of resources and energy to task execution, thereby improving the task completion rate. However, the other two algorithms are less flexible in resource utilization and task allocation, thus they are unable to fully utilize the available resources to meet task demands, leading to a lower task completion rate.

## 6. Conclusions and Future Works

This paper introduces a predictive offloading algorithm, LDLPSO, based on the Lyapunov approach, optimized for charging scenarios. Even in environments with highly random data leading to a relatively low prediction success rate, comparative experiments revealed that the LDLPSO algorithm still outperformed the other four algorithms in terms of the task completion rate and energy consumption. Specifically, compared to the LPSO algorithm, we achieved an improvement of approximately 25% in response speed at the cost of sacrificing some Fog Server (FS) performance. The overall average energy consumption was reduced by approximately 9.44%, demonstrating good scalability. However, there are still some limitations and areas for improvement. We did not consider the mobility of user nodes or adequately address issues related to cloud collaboration. In real scenarios, user nodes may move to different locations, and the frequent changes in users may impact the prediction, allocation, and resource utilization of tasks. Therefore, in future research, we need to consider how to adapt to the mobility of user nodes to enhance the algorithm’s performance further, making it better suited for diverse system heterogeneity and data heterogeneity in device scenarios.

In conclusion, although the LDLPSO algorithm in this paper achieved effective optimization results, further improvements are still necessary. Our future efforts will focus on researching the system heterogeneity and data heterogeneity of users, edge devices, and fog devices, leveraging the advantages of wireless charging networks. We aim to further enhance resource utilization efficiency, advance research in this field, and improve the performance and applicability of the algorithm. 

## Figures and Tables

**Table 1:** Comparisons of related works.

Paper	IoT-Fog ^1^	IoT-Cloud ^2^	Fog-Fog ^3^	Fog-Cloud ^4^	Dynamic	Energy	Prediction
[10]	✔			✔	✔	✔	
[13,14,18]	✔			✔			
[15]		✔				✔	
[16,19,20,24,25]	✔			✔		✔	
[17]	✔		✔			✔	
[21,22]	✔			✔	✔		
[23]	✔		✔		✔	✔	
[2]	✔			✔			✔
[1]	✔			✔	✔	✔	✔
Ours	✔		✔		✔	✔	✔

Note: “IoT-Fog” ^1^ means offloading from IoT devices to Fog, “IoT-Cloud” ^2^ means offloading from IoT devices to Cloud, “Fog-Fog” ^3^ means offloading between Fog tiers, while “Fog-Cloud” ^4^ means offloading from Fog to Cloud.

**Table 2:** List of symbols.

Symbol	Description
I,S,C,	*I* for IoT device or edge server, *S* for fog server, *C* for fog processor node (CFCN).
$F_{k},F_{S},F_{n}$	Computing resources for devices $I_{k}$, $F_{S}$, and $C_{n}$.
K	Number of IoT nodes.
s	Number of fog server nodes.
N	Set of fog computing nodes (CFCNs).
$F_{k,n}\left(t\right)$	The computed power assigned to $I_{k}$ by device $C_{n}$ at time slot *t*.
$F_{k,s}\left(t\right)$	The computed power assigned to $I_{k}$ by device $F_{S}$ at time slot *t*.
$\tau_{k}$	Transmission delay.
$Q^{R}\left(t\right)$	The actual task queue at time slot *t*.
$A_{k,w}\left(t\right)$	Prediction data for device when prediction window is *w* for device $I_{k}$ at time slot *t*.
$\tau_{t}$	Delay at time slot *t*.
$A_{k}\left(t\right)$	Prediction data for device for device $I_{k}$ at time slot *t*.
$W_{k}$	Prediction Window Size of $F_{s}$ for device $I_{k}$.
τ	Each time slot of its wireless channel.
$\lambda_{r}^{s}\left(t\right)$	Tasks actually arriving at $F_{s}$ at time *t*.
$\mu_{r}\left(t\right)$	The task delivered by $F_{s}$ at moment *t*.
$U_{r}\left(t\right)$	Tasks that time out at moment *t* on $F_{s}$.
$P_{s}$	Consumption of fog server node.
$\lambda_{k}\left(t\right)$	Arrival rate of tasks from $I_{k}$ for time slot *t*.
σ	Standard deviation of the calculated task volume for device $I_{k}$.
$D_{i}$	Average task size for device $I_{k}$.
$C_{j}$	Downstream bandwidth of device $F_{S}$.
$L_{s}$	Local processing task latency.
P(t)	Total energy consumption of time slot *t*.
$P_{n}\left(t\right)$	Energy consumed by fog node $P_{n}$ at slot *t*.
$P_{s}\left(t\right)$	Consumption of fog server node at slot *t*.
$P_{k,j}^{c}$	Energy consumption of device $I_{k}$ to transfer tasks to $F_{S}$.
$F_{k}^{c}$	Transmit power of device $I_{k}$.
$C_{k,j}$	Transmission rate from device $I_{k}$ to $F_{S}$.
α	Offload rate.
*w*	Inertial weighting of PSO.

## Data Availability

Data are simulation realizations, already described in the text, no additional datasets available.
